# Expression of both *Arabidopsis* γ-tubulin genes is essential for development of a functional syncytium induced by *Heterodera schachtii*

**DOI:** 10.1007/s00299-018-2312-7

**Published:** 2018-06-12

**Authors:** Elżbieta Różańska, Weronika Czarnocka, Łukasz Baranowski, Jakub Mielecki, Janice de Almeida Engler, Mirosław Sobczak

**Affiliations:** 1 Department of Botany, Faculty of Agriculture and Biology, Warsaw University of Life Sciences-SGGW, Nowoursynowska 159, 02-776 Warsaw, Poland; 2 Department of Plant Genetics, Breeding and Biotechnology, Faculty of Horticulture, Biotechnology and Landscape Architecture, Warsaw University of Life Sciences-SGGW, Warsaw, Poland; 3 INRA, Université Côte d’Azur, CNRS, ISA, Nice, France

**Keywords:** γ-Tubulin, Plant cytoskeleton, Cyst nematode, Nematode development

## Abstract

**Key message:**

After initial up-regulation, expression of *TUBG1* and *TUBG2* is significantly down-regulated in mature syncytia, but lack of expression of either of γ-tubulin genes reduces numbers of nematode infections and developing females.

**Abstract:**

Infective second stage juveniles of sedentary plant parasitic nematode *Heterodera schachtii* invade the root vascular tissue and induce a feeding site, named syncytium, formed as a result of cell hypertrophy and partial cell wall dissolution leading to a multinucleate state. Syncytium formation and maintenance involves a molecular interplay between the plant host and the developing juveniles leading to rearrangements and fragmentation of the plant cytoskeleton. In this study, we investigated the role of two *Arabidopsis* γ-tubulin genes (*TUBG1* and *TUBG2*), involved in MTs nucleation during syncytium development. Expression analysis revealed that both γ-tubulin’s transcript levels changed during syncytium development and after initial up-regulation (1–3 dpi) they were significantly down-regulated in 7, 10 and 15 dpi syncytia. Moreover, TUBG1 and TUBG2 showed distinct immunolocalization patterns in uninfected roots and syncytia. Although no severe changes in syncytium anatomy and ultrastructure in *tubg1-1* and *tubg2-1* mutants were observed compared to syncytia induced in wild-type plants, nematode infection assays revealed reduced numbers of infecting juveniles and developed female nematodes in mutant lines. Our results indicate that the expression of both *TUBG1* and *TUBG2* genes, although generally down-regulated in mature syncytia, is essential for successful root infection, development of functional syncytium and nematode maturation.

**Electronic supplementary material:**

The online version of this article (10.1007/s00299-018-2312-7) contains supplementary material, which is available to authorized users.

## Introduction

Sedentary plant endoparasitic nematodes infect a wide range of economically important crops causing serious losses to global agriculture (Chitwood 2003). Significant changes in root anatomy are caused by cyst forming nematodes, with two major genera: *Heterodera* and *Globodera*, that induce the formation of a feeding site, named syncytium, within the root vascular cylinder, which nourishes the nematode until the end of its life cycle. A syncytium is formed after partial cell wall dissolution between enlarging cells adjacent to the nematode head and it becomes subsequently multinucleate via protoplast fusion (Jones and Northcote 1972; Golinowski et al. 1996). During syncytium development, its cytoplasm becomes electron-dense, the central vacuole is replaced by numerous small ones and numbers of mitochondria, plastids and structures of endoplasmic reticulum increase. Syncytium development is regulated by a sophisticated cross-talk between nematode and plant via secreted nematode effectors that interact with various components of the host molecular regulatory pathways (Mitchum et al. 2013; Shukla et al. 2016). Moreover, the host plant cytoskeleton fragmentation and rearrangements have been reported during nematode parasitism, and possibly it is a target for secreted nematode effectors (de Almeida Engler et al. 2004). The cytoskeleton is a network of interconnected filamentous protein polymers, which gives structural stability to the cytoplasm and plays crucial functions in a number of cellular processes that are essential for plant morphogenesis, organogenesis and development (Takemoto and Hardham 2004; Wasteneys and Yang 2004). It is also an important component of the plant’s defence mechanism against abiotic and biotic stresses involving reorganization of cytoskeleton arrays that provide tracks for the delivery of molecules to their sites of action (Takemoto and Hardham 2004; Wasteneys and Yang 2004; Hardham 2013). Both microtubules (MTs) and actin filaments that are two key components of the eukaryotic cytoskeleton, are often co-aligned thus the specific role of each particular element is difficult to assign (Kost et al. 1999; Mayer and Jürgens 2002). MTs are highly dynamic polar polymers of non-covalently bound α-β-tubulin heterodimers that polymerize and depolymerize by de novo biosynthesis. Heterodimers are oriented with β-tubulin pointing toward the fast polymerizing plus (+) end and α-tubulin pointing toward the slowly polymerizing minus (−) end of the MT (Mayer and Jürgens 2002). MT polymerization is initiated at MT organizing centres (MTOCs) that act in centrosomes in fungal and animal cells (Wiese and Zheng 2006). In plants, MT nucleation in MTOCs is dispersed throughout the cytoplasm (Liu et al. 1994). γ-Tubulin proteins are known to be key molecular players in MT nucleation process. Plant γ-tubulin associates along MT arrays in a punctuate manner and is not restricted to MT minus end, which would be expected for a protein involved in MT nucleation (Binarová et al. 2000; Pastuglia et al. 2006). In *Arabidopsis*, there are two γ-tubulin family members (TUBG1 and TUBG2) (Pastuglia et al. 2006). *TUBG1* and *TUBG2* gene’s coding regions are very similar, exhibiting 95% nucleotide sequence identity (Liu et al. 1994). Both polypeptides consist of 474 amino acids and share 98.1% identity and 99.4% similarity according to BLAST analysis. Both γ-tubulin genes are constitutively expressed at high levels in all plant organs (Zimmermann et al. 2004). *Arabidopsis* insertional T-DNA mutants, *tubg1-1* and *tubg2-1*, have a wild-type phenotype in terms of growth, development and fertility (Binarová et al. 2000; Pastuglia et al. 2006). The absence of phenotypic changes in both single mutants indicates high, if not complete, functional redundancy between TUBG1 and TUBG2 genes in *Arabidopsis*. Double *tubg1-1*/*tubg2-1* mutant is lethal (Pastuglia et al. 2006).

MTs play important roles in a number of growth and developmental processes, including chromosome movement, cytokinesis and the orientation of cellulose microfibrils in the plant cell wall (Fisher and Cyr 1998). Liu et al. (1994) have shown that in plant protoplasts MTs are generated at various cellular locations, such as nuclear envelope or the inner face of the plasma membrane region. Major rearrangements of the plant cytoskeleton occur also during nematode feeding cell development (de Almeida Engler et al. 2004; Banora et al. 2011). In syncytia both the MT and actin cytoskeletons are fragmented, although structured cortical MTs close to the plasma membrane seem still present. Studies of the interaction between *Arabidopsis* and the root-knot nematode *Meloidogyne incognita* reported the requirement of γ-tubulin complexes for giant cell development and their essential function in remodelling of MT network in giant cells, but also in successful nematode reproduction (Banora et al. 2011). Therefore, we questioned if and where γ-tubulins are present in the syncytium, a type of nurse cells ontogenetically different from the giant cells in spite of their ultrastructural similarity, and if these cytoskeletal proteins play a role in syncytia development and maintenance. In the current study, we demonstrated that after initial increase in the expression of *TUBG1* and *TUBG2* genes at early stages of syncytium development, their expression was significantly decreased in the mature syncytia, compared to uninfected roots. Ultrastructural analyses and γ-tubulin immunolocalization in syncytia induced in wild type and *tubg1-1* and *tubg2-1* mutants showed only minor cytological and anatomical differences. However, in spite of apparent down-regulation of *TUBG1* and *TUBG2* expression in mature syncytia, the nematode infection and development tests performed on *tubg1-1* and *tubg2-1* mutants showed that the lack of expression of any of γ-tubulin genes significantly decreased numbers of infecting juveniles and maturing nematode females whereas the development of males was uninfluenced. It suggests that generally accepted functional redundancy of TUBG1 and TUBG2 (Pastuglia et al. 2006) in the case of formation and maintenance of cyst nematode-induced syncytium as well as nematode development is limited.

## Materials and methods

### Plant growth condition and nematode inoculation

Sterile seeds of wild-type *Arabidopsis thaliana* (L.) Heynh. ecotype Columbia (Col-0), γ-tubulin knock-out mutant lines *tubg1-1* (with T-DNA insert in the first exon of *TUBG1* associated with 55-bp deletion in the coding region) and *tubg2-1* (with fully deleted *TUBG2* coding sequence) were germinated and grown on Knop medium under 16/8-h light/dark photoperiod at 25 °C (Sijmons et al. 1991). Two-week-old seedlings were inoculated with 70 freshly hatched second stage juveniles (J2) of *Heterodera schachtii* Schmidt per plant, obtained from sterile agar stock cultures. Inoculated plants were kept in a growth chamber (Labudda et al. 2016). Progress of nematode infection was monitored in vitro during the first 3 days after inoculation using a stereo microscope, thus invasion time was precisely assessed. Root segments containing syncytia were collected at 1, 3, 5, 7, 10 and 15 days post infection (dpi). Pieces of uninfected roots were collected at the corresponding time points and root zones from non-inoculated plants.

## RNA isolation and cDNA synthesis

Total RNA was isolated from root segments containing syncytia collected from wild-type *A. thaliana* ecotype Col-0 plants and γ-tubulin knock-out mutants: *tubg1-1* and *tubg2-1* at 1, 3, 5, 7, 10 and 15 dpi, and corresponding samples of uninfected roots. Nematodes were removed from samples before freezing. Total RNA was isolated using GeneMATRIX Universal RNA Purification Kit (EURx, Gdansk, Poland) with additional step of on-column DNase I treatment. RNA concentration, purity and integrity were tested spectrophotometrically with Nanodrop 2000 (Thermo Fisher Scientific, Waltham, MA, USA) or after electrophoretic separation in 1% (w/v) agarose gels in 1× TBE running buffer, they were visualized by SimplySafe (EURx) staining and photographed. After equalization of RNA concentrations, cDNA was synthesized using High Capacity cDNA Reverse Transcription Kit (Thermo Fisher Scientific).

## Quantitative real-time PCR

Intron-spanning primers (Supporting Information Table S1) were designed with the Universal Probe Library Assay Design Center Probe Finder software (Roche; http://www.roche-applied-science.com/). AT5G10790 gene coding ubiquitin-specific protease 22 (UBP22), which demonstrated the most stable expression in RefSeq tool search within Genevestigator database (Hruz et al. 2008), was used as the endogenous reference. qPCR reactions were performed in 96-well plates using CFX96 Touch™ Real-Time PCR Detection System (Bio-Rad, Hercules, CA, USA) according to the manufacturer’s instruction. 4 µl of 1:25 diluted first-strand cDNA was used as a template. Apart from cDNA, each reaction contained 7.5 µl of iTaq Universal SYBR Green Supermix (Bio-Rad), 0.3 µl of each primer (final concentration 0.2 µM) and 2.9 µl of sterile water. Reaction conditions are shown in Supporting Information Table S2. Expression of each gene was tested in two biological replicates and three technical repetitions. The specificity of amplified PCR products was verified by melting curve analysis. For statistical analysis, the calculation of reaction efficiency was performed using LinRegPCR software (Ramakers et al. 2003) whereas the absolute normalised gene expression levels and statistical significance of their differences was calculated using REST2009 software (Pfaffl et al. 2002).

## Genotyping

For genotyping of wild type and mutant plants 1 µl of 0.5 µg µl^−1^ first-strand cDNA was used as a template in PCR. Apart from cDNA, each reaction (final volume of 25 µl) contained 10 mM of each gene-specific primer (Supporting Information Table S3), 0.2 mM dNTPs, 1× reaction buffer, and 1.25 U DreamTaq Green DNA polymerase (Fermentas/Thermo Scientific) and sterile water. Reaction conditions are given in Supporting Information Table S4. A negative control was run without cDNA template. Amplified fragments were electrophoresed on a 1.2% (w/v) agarose gels in 1× TBE running buffer, visualized by SimplySafe (EURx) and photographed. Expression of each gene was tested in three biological replicates.

## Infection test

Twelve days after germination, 50 seedlings of wild-type *A. thaliana* ecotype Col-0 and γ-tubulin knock-out mutants: *tubg1-1* and *tubg2-1* were inoculated in vitro with 70 surface-sterilized freshly hatched J2s of *H. schachtii* per plant. Infected seedlings were kept at 20 °C under 16/8-h light/dark photoperiod. Infection sites found with a stereo microscope were counted at 5 and 15 dpi, when infecting juveniles were well-established in roots, and females and males could be clearly discriminated, respectively. Infection assays were performed in three biological replicates. For the statistical analysis of the results Tukey’s multiple comparison test was performed using GraphPad Prism 6 software.

## Anatomic and ultrastructural analysis

Uninfected roots and root segments containing 3, 7 and 15 dpi syncytia were dissected and processed for microscopic examinations as described by Golinowski et al. (1996). Light and transmission electron microscopy analyses were conducted on sections obtained from the same samples. Root segments were serially sectioned on a Leica RM2165 microtome (Leica Microsystems, Wetzlar, Germany) into 3-µm thick sections that were collected on glass slides, stained with 1% (w/v) aqueous solution of crystal violet dye (Sigma-Aldrich, St. Louis, MO, USA) and examined in an Olympus AX70 ‘Provis’ light microscope (Olympus, Tokyo, Japan) equipped with an Olympus DP50 digital camera (Olympus). At selected places, ultrathin sections (90 nm thick) were taken for transmission electron microscopy with a Leica UCT ultramicrotome (Leica Microsystems). Ultrathin sections were stained with saturated solution of uranyl acetate (Sigma-Aldrich) followed by incubation in lead citrate (Sigma-Aldrich) and examined in an FEI 268D ‘Morgagni’ transmission electron microscope (FEI Company, Hillsboro, OR, USA) equipped with an Olympus-SIS ‘Morada’ digital camera (Olympus). Samples were collected in two independent experiments and at least three randomly selected syncytia per each experiment, time point and genotype were serially sectioned for microscopic examinations.

## Immunolocalization of γ-tubulin

Segments of roots from non-inoculated plants and roots containing syncytia were collected from wild-type *A. thaliana* ecotype Col-0 plants and γ-tubulin *tubg1-1* and *tubg2-1* knock-out mutants at 3, 7 and 15 dpi. They were fixed in 3.7% (w/v) paraformaldehyde in MSB buffer (50 mM PIPES, 5 mM EGTA, 5 mM MgSO_4_, 10% (v/v) DMSO; pH 6.9) for 2 h and dehydrated in a graded ethanol series [10, 30, 50 and 70% (v/v)] diluted with MSB supplemented with 10 mM DTT for 30 min at 4 °C. Further dehydration steps were performed with 96% (v/v) and 100% ethanol containing 10 mM DTT for 1 h at 4 °C per step. After dehydration the samples were infiltrated with a graded series of butyl–methyl–methacrylate resin [Sigma-Aldrich; 80% (v/v) butyl methacrylate, 20% (v/v) methyl methacrylate and 0.5% (w/v) benzoilethyl ether] mixed with 100% ethanol (v/v mixtures 3:1, 1:1, 1:3) for 2 h per step and finally infiltrated with pure resin for 6 h. Samples were transferred to BEEM embedding capsules (Polyscience, Niles, IL, USA) filled up with fresh butyl–methyl–methacrylate resin. The resin was polymerized at − 20 °C for 20 h under long wave UV illumination (Gubler 1989; Baskin et al. 1992; de Almeida Engler et al. 2001; Banora et al. 2011). Samples were sectioned on a Leica RM2165 microtome (Leica Microsystems) and sections (3-µm thick) were collected on poly-l-lysine-coated glass slides (Menzel-Gläser, Braunschweig, Germany). The resin was removed from the sections after 5 and 10 min stirring in acetone at room temperature. The slides were transferred to 100% ethanol, then rehydrated in descending ethanol series [70, 50, and 30% (v/v)] and transferred to 10 mM PBS for 15 min. The procedure of immunolocalization followed the method described by Baranowski et al. (2018). Monoclonal mouse anti-γ-tubulin antibody (cat. no T6557; Sigma-Aldrich) diluted 1:100 was used as primary antibody that was detected with secondary goat anti-mouse IgG antibody coupled with AlexaFluor Plus 488 (Invitrogen, Carlsbad, CA, USA) diluted 1:200. In control labelling, primary antibody was omitted and sections were incubated in a blocking solution. After three washes in PBS supplemented with 0.05% (v/v) Tween 20 in the darkness for 10 min, samples were counterstained with 1 µg ml^−1^ DAPI (4′,6-diamidino-2-phenylindole; Sigma-Aldrich) for 10 min. Slides were then washed twice with sterile water and mounted in ImmunoFluore Mounting Medium (ICN Biomedicals, Irvine, CA, USA). They were examined under an Olympus AX70 ‘Provis’ epifluorescence microscope equipped with a UM61002 fluorescence filter set. Samples were collected in two independent experiments and at least three randomly selected syncytia per each experiment, time point and genotype were sectioned and subjected for immunolocalization assays.

## Immunoblotting

To confirm specificity of commercially available monoclonal mouse anti-γ-tubulin antibody (cat. no T6557; Sigma-Aldrich) against *Arabidopsis* γ-tubulins total proteins were isolated from liquid nitrogen frozen roots of wild-type *A. thaliana* ecotype Col-0 plants and γ-tubulin *tubg1-1* and *tubg2-1* knock-out mutants grown for 14 days on Knop medium under conditions described above. Proteins were isolated with 2× Laemmli Sample Buffer (Bio-Rad). Total protein concentration was determined with RC DC Protein Assay (Bio-Rad). 20 µg of total protein extract was separated on 8% polyacrylamide gel by sodium dodecyl sulphate polyacrylamide gel electrophoresis (SDS-PAGE) with β-mercaptoethanol as a reducing agent and transferred onto nitrocellulose membrane 0.45 µm (Bio-Rad). Membranes were blocked in 1% (v/v) Blotting-Grade Blocker (Bio-Rad) in 25 mM Tris-HCl (pH 7.6), 150 mM NaCl and 0.05% (v/v) Tween 20 (TBS-T) for 1 h at RT. Membranes were incubated overnight at 4 °C with 1:5000 diluted primary antibody, washed with TBS-T and incubated with 1:5000 diluted rabbit anti-mouse IgG H&L (HRP) secondary antibody (cat. no ab97046; abcam, Cambridge, United Kingdom). After incubation, membranes were washed for three times in TBS-T; chemiluminescence was detected using the Pierce™ ECL Plus Western Blotting Substrate (Thermo Fisher Scientific) and ChemiDoc Imaging Systems (Bio-Rad).

## Results

### *TUBG1* and *TUBG2* expression is down-regulated in syncytia

**Fig. 1:**
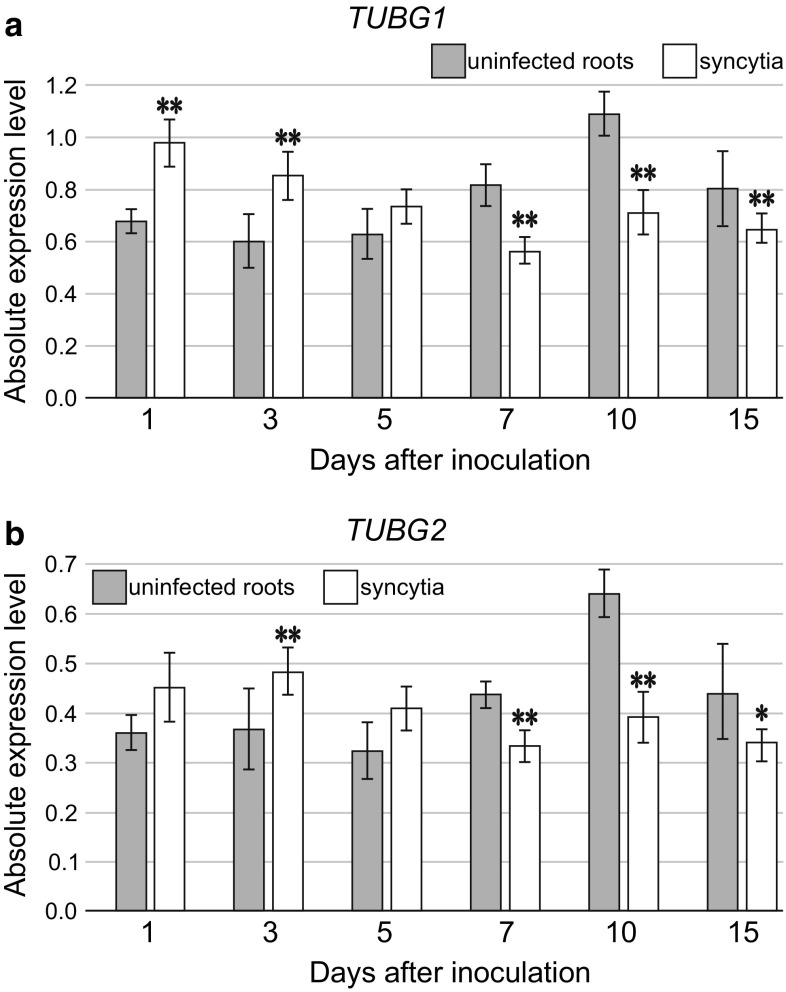
The analysis of changes in *TUBG* expression levels. Absolute normalised levels of *TUBG1* (**a**) and *TUBG2* (**b**) transcripts in syncytia at 1, 3, 5, 7, 10 and 15 dpi (white) in comparison to non-inoculated *Arabidopsis* roots (grey). Statistical analysis was performed using LinRegPCR (calculation of reaction efficiency) and REST2009 (calculation of  absolute normalised gene expression level and statistical significance of their differences). Expression level for each *TUBG* was normalised to the endogenous control. Bars represent mean values ± SE (*n* = 6). Asterisks above the bars indicate statistically significant differences in comparison to the non-inoculated roots at *p* < 0.05 (*) or *p* < 0.005 (**)

To obtain expression profiles of *TUBG1* and *TUBG2* genes encoding γ-tubulins, reverse transcription quantitative PCR (qPCR) was performed. The total RNA was extracted from root samples containing early stage (1 and 3 dpi), intermediate stage (5, 7 and 10 dpi) and mature syncytia (15 dpi). Samples of uninfected *Arabidopsis* Col-0 roots collected at corresponding time points were used as controls. Absolute normalised expression of *TUBG1* and *TUBG2* in uninfected roots revealed similar pattern of expression changes for both genes (Fig. 1). Their expression remained relatively stable till 5 dpi. Then it increased reaching maximum in samples corresponding to 10 dpi syncytia. In roots corresponding to 15 dpi syncytia the expression of *TUBG1* and *TUBG2* decreased again. In syncytia, the absolute normalised expression of both γ-tubulin genes changed following similar pattern (Fig. 1). Generally, it was elevated in early stage of syncytia development in comparison to corresponding uninfected root samples. In the case of *TUBG1* this difference was statistically significant at 1 and 3 dpi, whereas for *TUBG2* the increase was significant only at 3 dpi. In 7, 10 and 15 dpi syncytia the absolute normalised expression of both *TUBG* genes decreased and was significantly lower than in corresponding samples collected from non-inoculated roots. These results indicate that the expression level of both genes encoding γ-tubulins is generally up-regulated at early stages of infection when juveniles establish their feeding sites and successively down-regulated in well-established syncytia at 7, 10 and 15 dpi (Fig. 1).

### Lack of one of γ-tubulins leads to lower numbers of infection sites and developed nematode females

**Fig. 2:**
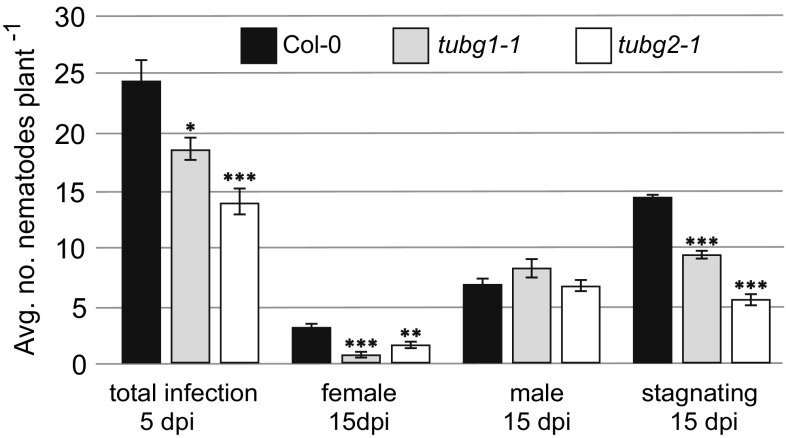
Nematode infection tests carried out on γ-tubulin mutant lines (*tubg1-1* and *tubg2-1*) and wild-type plants. Bars represent mean values ± SE (*n* = 50). Statistical analysis was carried out by ANOVA Tukey’s test. Asterisks above the bars indicate statistically significant differences in comparison to the uninfected roots at *p* < 0.05 (*), *p* < 0.005 (**) or *p* < 0.001 (***)

Before carrying out any experiments on *tubg1-1* and *tubg2-1* mutants (Pastuglia et al. 2006), their homozygosity was first confirmed by PCR (Supporting Information Fig. S1). To determine whether the lack of either γ-tubulin protein influences the infection process as well as syncytium and nematode development, wild-type Col-0 and *tubg1-1* and *tubg2-1* mutant plants were inoculated with 70 J2s of *H. schachtii* per plant. Five days after inoculation the number of infection sites was significantly lower in both γ-tubulin mutant lines, reaching about 75% for *tubg1-1* and 57% for *tubg2-1* of the average infection number in the wild-type plants (Fig. 2). The number of developed female nematodes within the mutant roots at 15 dpi was significantly reduced in both *tubg1-1* and *tubg2-1*, to about 22 and 51%, respectively, compared to the wild-type plants, whereas the differences in the numbers of developed males were statistically insignificant. There were no statistically significant differences between *tubg1-1* and *tubg2-1* mutants in the number of infection sites, females or males. The number of underdeveloped or stagnating juveniles at 15 dpi was significantly lower for both γ-tubulin mutants in comparison to wild-type plants. Interestingly, the percentage of underdeveloped and stagnating juveniles at 15 dpi in relation to average number of infections at 5 dpi was the highest in wild-type plants (59%), whereas only 51 and 40% of invading juveniles were underdeveloped or stagnating at 15 dpi in *tubg1-1* and *tubg2-1* roots. These results strongly indicate that both TUBG1 and TUBG2 are especially important for effective nematode infection, but also later on they have fundamental importance for development of female nematodes.

### Anatomy and timing of syncytium development differ between wild type and γ-tubulin mutants

**Fig. 3:**
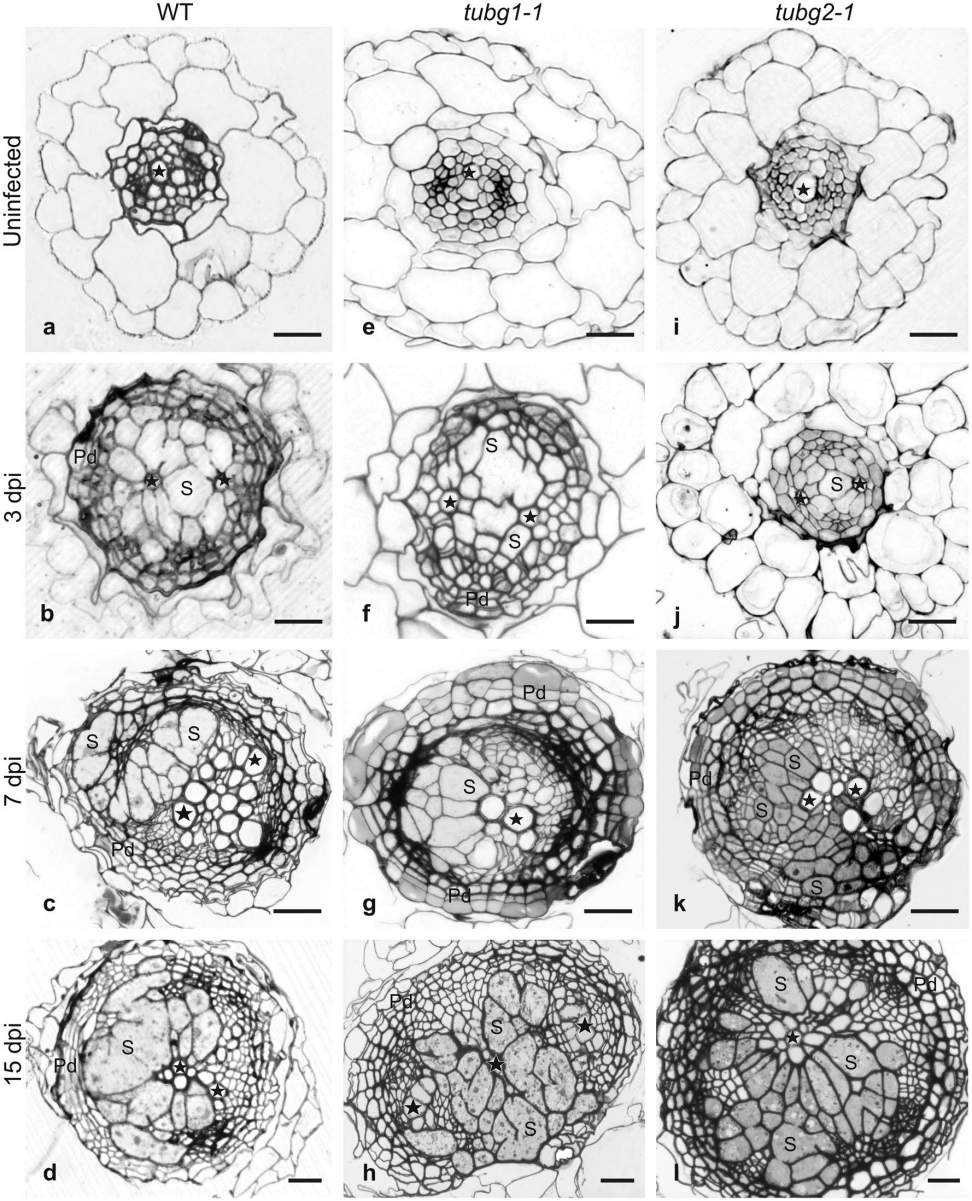
Development of nematode-induced syncytia in wild type and *TUBG* mutant lines. Bright field light microscopy images of cross sections taken from wild type (**a**–**d**), *tubg1-1* (**e**–**h**) and *tubg2-1* (**i**–**l**) roots. Sections of uninfected roots were taken at root-hair zone (**a, e** and **i**). Sections of syncytia at 3 dpi (**b, f** and **j**) were taken close to the nematode heads whereas sections of 7 dpi (**c, g** and **k**) and 15 dpi (**d, h** and **l**) were taken at the widest part of syncytia. *S* syncytium, *Pd* periderm. Asterisks indicate xylem vessels. Scale bars 20 µm

To investigate anatomical changes induced in wild-type Col-0, *tubg1-1* and *tubg2-1* lines upon cyst nematode infection, we analysed serial cross sections taken through uninfected roots and the roots containing 3, 7 and 15 dpi syncytia (Fig. 3). No differences were observed in root development or in tissue organization of uninfected roots between wild type (Fig. 3a) and γ-tubulin mutant lines (Fig. 3e, i). Although syncytia developing in the wild type and in both mutant lines were anatomically similar, a couple of minor differences appeared. In general, anatomy of syncytia induced in *tubg1-1* mutant was more similar to syncytia induced in wild-type plants (Fig. 3b–d, f–h) than to those induced in *tubg2-1* roots (Fig. 3j–l). In *tubg2-1*, a delay in syncytium expansion was noticeable at 3 dpi (Fig. 3j versus b, f). Syncytia in *tubg2-1* were smaller and additionally swollen root hairs were visible at infected root regions as demonstrated also for galls (Banora et al. 2011). Weaker syncytial element hypertrophy, less cell fusion and fewer cell wall openings were usually found in 7 and 15 dpi syncytia induced in *tubg1-1* and *tubg2-1* mutants (Fig. 3c, d, k, l) compared to syncytia induced in control plants (Fig. 3g, h). In parallel to syncytium development, pericyclic and cambial cells start to divide and to form periderm and secondary conducting tissues in all examined genotypes. Similarly to syncytia, development of the secondary tissues seemed to be delayed in both γ-tubulin mutants in comparison to wild-type plants at 3 dpi (Fig. 3j, f versus b). However, at 7 dpi the number of periderm cells and number of periderm cell layers were similar in all three genotypes (Fig. 3c, g, k). At 15 dpi the periderm was better developed around syncytia induced in both γ-tubulin mutant lines (Fig. 3h, l) than in wild-type roots (Fig. 3d).

**Fig. 4:**
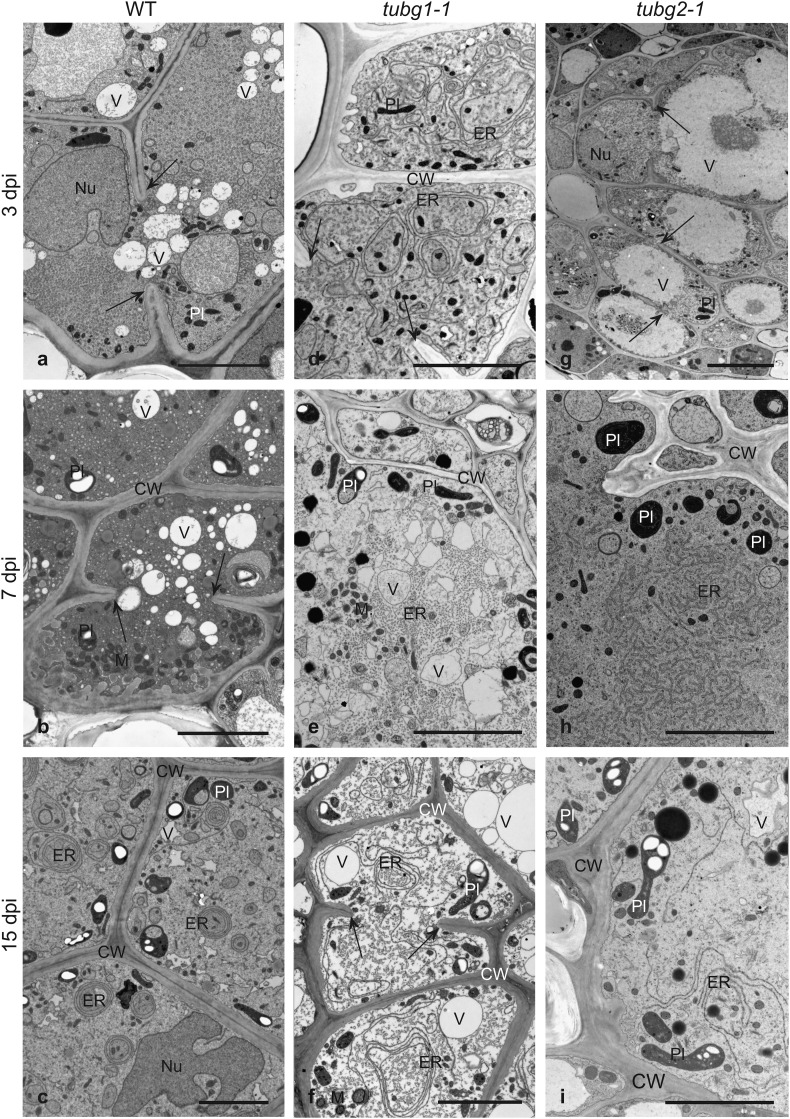
Transmission electron microscopy micrographs of cross sections of syncytia induced in wild type and *TUBG* mutant lines. Sections were taken through syncytia induced in wild-type plants (**a**–**c**), *tubg1-1* (**d**–**f**) and *tubg2-1* (**g**–**i**) and collected at 3 dpi (**a, d** and **g**), 7 dpi (**b, e** and **h**) and 15 dpi (**c, f** and **i**). *CW* cell wall, *ER* endoplasmic reticulum, *M* mitochondrion, *Nu* nucleus, *Pl* plastid, *V* vacuole. Arrows indicate cell wall stubs flanking cell wall openings. Scale bars 5 µm

The ultrastructural organization of syncytia induced in the wild type and both γ-tubulin mutant lines showed profuse organelles and highly electron-dense cytoplasm (Fig. 4). In contrast to syncytia induced in wild-type plants, in syncytia formed in *tubg1-1* and *tubg2-1* mutants the cisternae of endoplasmic reticulum (ER) proliferated strongly and they were often arranged into circular swirls indicating that the lack of either γ-tubulin might influence ER morphology (Fig. 4d, f, h, i). Nuclei in syncytia were hypertrophied and lobed (Fig. 4a, c, g) and numerous plastids and mitochondria were located close to the syncytial cell walls, especially at 7 and 15 dpi (Fig. 4). The syncytial cell walls were typically thickened, but cell wall openings varied in sizes among genotypes (Fig. 4a, b, d, f, g). They were limited in size and number in syncytia induced in the *tubg2-1* line indicating a delay in cellular fusion (Figs. 3j, k, l, 4g). At 7 dpi, in γ-tubulin mutant lines, higher number of enlarged plastids appeared compared to the wild type (Fig. 4e, h). They became filled with starch grains in mature syncytia at 15 dpi (Fig. 4f, i versus b and c). Features of cellular degradation such as osmiophilic, flocculent or translucent cytoplasm became visible in syncytia since 7 dpi in both γ-tubulin mutants (Fig. 4e, f, h, i) and were still not found in syncytia induced in wild-type roots at 15 dpi (Fig. 4c).

### Localization of TUBG1 and TUBG2 differs in uninfected roots and syncytia

**Fig. 5:**
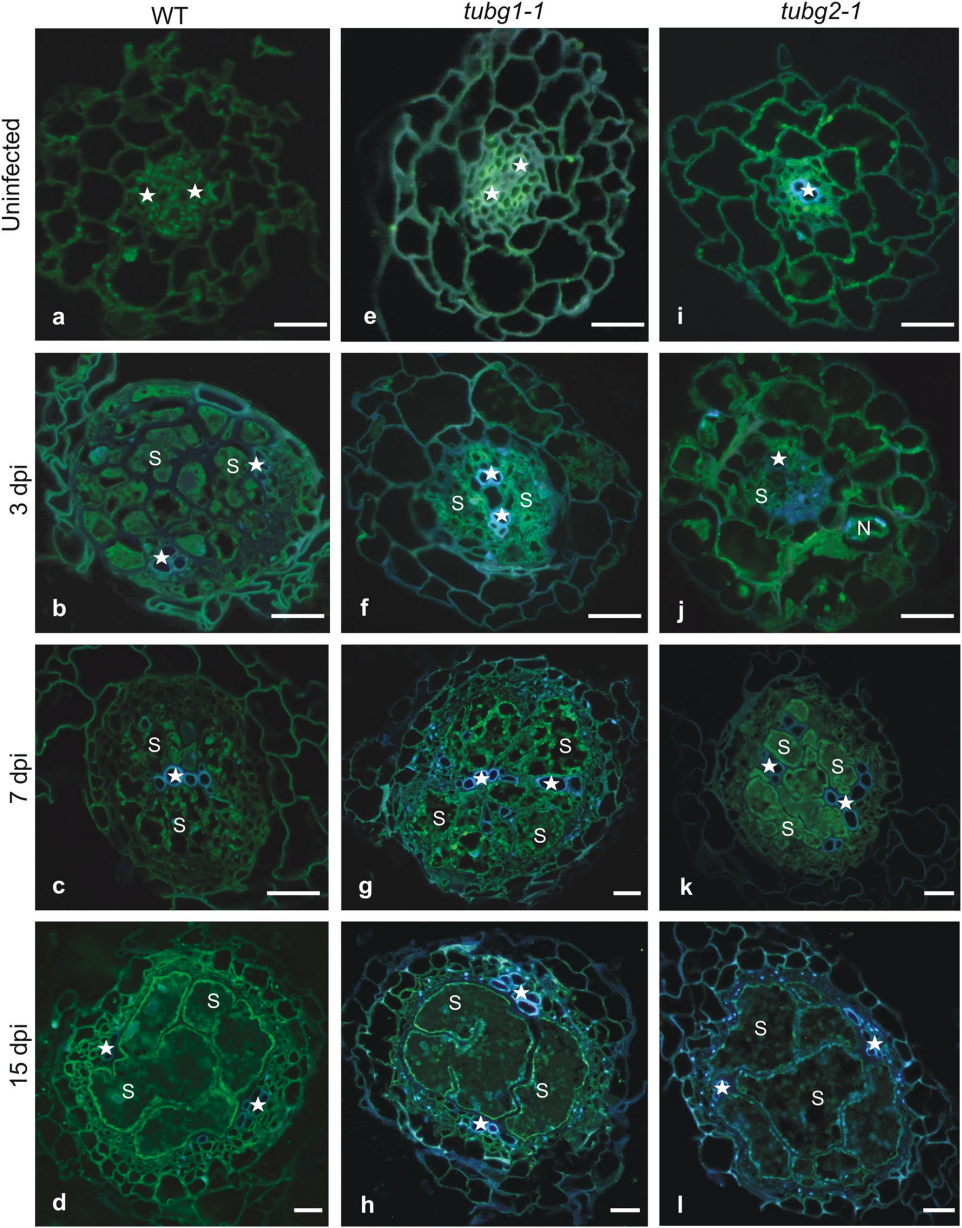
Immunohistochemical localization of γ-tubulin in uninfected roots and in roots containing nematode-induced syncytia. Epifluorescence micrographs of cross sections taken from wild-type (**a**–**d**), *tubg1-1* (**e**–**h**) and *tubg2-1* (**i**–**l**) mutant lines. Sections of uninfected root of wild type (**a**), *tubg1-1* (**e**) and *tubg2-1* (**i**) plants. Sections of 3 dpi syncytia induced in wild-type (**b**), *tubg1-1* (**f**) and *tubg2-1* (**j**) plants taken at the region close to the nematode head. Sections of 7 dpi syncytia induced in wild-type (**c**), *tubg1-1* (**g**) and *tubg2-1* (**k**) plants taken at the widest region of syncytium remote from nematode head. Sections of 15 dpi syncytia induced in wild-type (**d**), *tubg1-1* (**h**) and *tubg2-1* (**l**) plants taken in close to the nematode heads. Asterisks indicate xylem vessels. *S* syncytium. Silver–blue autofluorescence of tracheal elements and blue fluorescence of nuclei stained with DAPI (**e**–**l**). Scale bars 20 µm

The immunolocalization of TUBG1 and TUBG2 proteins (Fig. 5) in sections of uninfected roots and syncytia induced in the wild type and in *tubg1-1* and *tubg2-1* mutant lines was conducted using commercially available monoclonal antibody. Amino acid sequence of TUBG1 shows 98% identity to TUBG2 (Supporting Information Fig. S2). The synthetic oligopeptide used for antiserum production consisted of 16 amino acids (amino acids 38–53) identical for both γ-tubulins (http://www.sigma-aldrich.com; Supporting Information Fig. S3). Therefore, the antibody detects both TUBG1 and TUBG2 proteins in mutant and wild-type *Arabidopsis* plants. To confirm antibody specificity we isolated total proteins from non-inoculated roots of wild-type and mutants plants and performed immunodetection assay (western blot) that detected single band of 58 kDa in all genotypes (Supporting Information Fig. S4). Thus, the antibody was used for immunolocalization of both γ-tubulins in wild-type roots and syncytia (Fig. 5a–d), immunolocalization of TUBG2 in *tubg1-1* mutant (Fig. 5e–h) and TUBG1 in *tubg2-1* mutant (Fig. 5i–l).

In wild-type uninfected roots and syncytia, γ-tubulins were present in the cytoplasm of all root cells and syncytial elements (Fig. 5a–d). At 15 dpi γ-tubulins were mainly observed in the cell cortex of syncytial elements (Fig. 5d) suggesting that this would be a region of MTs polymerization. TUBG2 was observed in uninfected *tubg1-1* mutant roots mainly in parenchymatic vascular cylinder cells (Fig. 5e) and TUBG1 overall in root cells of *tubg2-1* mutant (Fig. 5i). In young syncytia (3 dpi) induced in wild-type plants the fluorescence signal of γ-tubulins was present in the syncytial cytoplasm and in surrounding vascular cylinder and cortex cells (Fig. 5b). Developing syncytia in *tubg2-1* and *tubg1-1* mutant demonstrated similar localization of TUBG1 and TUBG2, respectively (Fig. 5j, f). Similar overall distribution of γ-tubulins was observed in wild-type and both γ-tubulin mutants in 7 dpi syncytia (Fig. 5c, g, k). However, the intensity of fluorescence coming from TUBG2 detection was higher in syncytium and surrounding vascular tissue in *tubg1-1* plants, compared to TUBG1 in *tubg2-1* plants (Fig. 5g versus k). Interestingly, γ-tubulin’s localization pattern changed in 15 dpi syncytia where γ-tubulins were mostly localized along the syncytial cell walls in the cytoplasm cortex and almost absent in other regions of syncytial cytoplasm (Fig. 5d, h, l). Moreover, TUBG2 was detected in regions close to syncytial nuclei and in the vascular cylinder cells surrounding the syncytium (Fig. 5h). These results indicate that the localization of both TUBG1 and TUBG2 is dependent on syncytium developmental stage.

## Discussion

A plenty of research in the field of plant–nematode interactions have been recently devoted to search for plant’s nematode susceptibility factors, namely, proteins whose presence or absence is fundamental for proper development of nematode-induced feeding sites (de Almeida Engler et al. 2005; Lilley et al. 2005; Shukla et al. 2016). Extensive changes in plant cytoskeleton organization in feeding sites seem to be good indication that at least some of the proteins associated with cytoskeleton can play such a role.

The plant cytoskeleton participates in many cellular processes such as cytodifferentiation, cell division, cytoplasm streaming, cell wall synthesis, organelle and protein trafficking, as well as early defence response (Boevink et al. 1998; Kost et al. 1999; de Almeida Engler et al. 2004; Johansson et al. 2014). The dynamics of the plant cytoskeleton is determined by the two interconnected types of filamentous components, MTs and actin filaments that cooperatively function during a variety of cellular processes. γ-Tubulins are essential for the formation and nucleation of MTs in plant cells. They localize in the vicinity of the plasma membrane and cell nuclei and are associated with all types of the MT arrays: interphase MTs, preprophase band, mitotic spindle and phragmoplast. Plant γ-tubulins are associated with protein complexes named MT organizing centres (MTOCs) that are anchored in membranes (Binarová et al. 2006; Liu et al. 1994). In the field of plant–nematode interaction, the role of cytoskeleton was widely examined (de Almeida Engler et al. 2004) and detailed analyses of γ-tubulin role in development and maintenance of giant cells induced by the root-knot nematode, *M. incognita*, were conducted (Banora et al. 2011).

Root-knot nematodes establish feeding sites in the root differentiation zone inducing cell hypertrophy and nuclear divisions without cytokinesis of selected group of host’s vascular cylinder cells. This process gives rise to large multinucleate feeding cells, termed giant cells that are surrounded by dividing cells forming gall (Williamson and Hussey 1996; de Almeida Engler et al. 1999; Williamson and Gleason 2003). Cyst nematodes, the second group of sedentary plant parasitic nematodes, establish their feeding sites by the formation of partial cell wall dissolutions between the initial syncytial cell and neighbouring cells, resulting in the development of syncytial type feeding site that becomes multinucleate via protoplasts fusion (Golinowski et al. 1996; Williamson and Hussey 1996; Williamson and Gleason 2003). The main aim of the current work was to elucidate if γ-tubulins and MTs, which formation depends on γ-tubulins, play similar roles in the development of syncytia induced by cyst nematodes as found in ontogenetically different giant cells induced by root-knot nematodes (Banora et al. 2011).

Our qPCR results indicated significantly increased expression of *TUBG1* in 1 and 3 dpi syncytia and *TUBG2* in 3 dpi syncytia, compared to non-infected roots at corresponding time points (Fig. 1). At 5 dpi the expression of both γ-tubulin genes was higher than in corresponding non-inoculated roots, although insignificantly. Thereafter, situation changed and the expression levels of *TUBG1* and *TUBG2* were significantly lower in syncytia between 7 and 15 dpi than in corresponding control roots. Such expression pattern is opposite to the results for giant cells, where elevated levels of γ-tubulins mRNA were shown between 7 and 21 dpi (Banora et al. 2011). However, lower *TUBG1* and *TUBG2* expression in well-established syncytia is in line with our present study showing less MTs assembly in syncytia at this stage. Moreover, both *TUBG1* and *TUBG2* were expressed in well-established syncytia at similar levels at each time point, which is in agreement with the results of transcriptome analyses showing no significant changes in expression levels of *TUBG1* and *TUBG2* in 5 and 15 dpi syncytia induced in *Arabidopsis* roots by *H. schachtii* (Szakasits et al. 2009). Surprisingly, in our study the significantly lower level of absolute normalised expression of *TUBG1* and *TUBG2* genes in well-established syncytia was not achieved due to their lower expression in syncytia themselves, but it was granted by elevated expression of both genes in control samples. To make comparisons between infected and uninfected roots we dissected pieces of uninfected roots at the similar distance from the root tips as syncytia were located. Roots of cyst nematode-infected plants usually do not stop to elongate. In contrast to uninfected roots collected between 1 and 5 dpi, those collected between 7 and 15 dpi had well-developed secondary state of growth (cortex and epidermis was shed and secondary conductive and cover tissues were developed). Thus, the elevated expression of both *TUBG1* and *TUBG2* genes at these time points can be attributed to abundant divisions of the vascular cylinder cells that demand formation of mitotic spindles and phragmoplasts composed of MTs. Periderm and secondary conductive tissues were formed also around syncytia in all three examined genotypes (Fig. 3). The lower levels of *TUBG1* and *TUBG2* expression in well-established syncytia indicate that either the number of dividing cells is lower or the level of γ-tubulin genes expression is strongly decreased. The former does not seem to be possible as cell divisions in the periderm occur around syncytia induced in both wild-type and γ-tubulin mutant’s roots. Thus, most probably, the decrease of *TUBG1* and *TUBG2* expression levels is due to strong decrease of their expression inside syncytia. The syncytia easily exceed the length of 2 mm at 10–15 dpi (Sobczak et al. 1997) and the expression of genes in syncytium and in cells directly abutting syncytium can differ dramatically (Fudali et al. 2008; Karczmarek et al. 2008). We realise that our results are strongly biased by signal originating from cells surrounding syncytium and that the best way to obtain data is to microaspirate syncytial protoplasts (Szakasits et al. 2009). Summarizing, it seems that up-regulation of expression of both *TUBG1* and *TUBG2* is of fundamental importance for successful root invasion and establishment of syncytium and both *TUBG1* and *TUBG2* genes play a role of nematode susceptibility factors.

Significantly lower numbers of invading juveniles found in both γ-tubulin mutants suggest that their roots are apparently less attractive for J2s than roots of wild-type plants. However, if the juveniles managed to infect roots, the number of underdeveloped, delayed in development or stagnating juveniles at 15 dpi is significantly lower in γ-tubulin mutant plants than in control plants (Fig. 2). One possible explanation for this phenomenon is lower content of available free γ-tubulin molecules that decrease dynamics of MTs assembly in mutant plants. Second, it may be related to incomplete redundancy of TUBG1 and TUBG2 that may specifically act in some secretory processes or processes related to cell wall organization that made roots of mutants plants less attractive to migratory J2s. Third, it has to be pin-pointed that beet cyst nematode is an amphimictic species and individual juveniles that form a population used in our study differ genetically (Sijmons et al. 1991). Thus, each of them may respond in individual specific way to plant secreted attractants and plant structural features that may be modified in γ-tubulin mutants in a yet unknown way.

Microscopic observations showed only a set of discreet structural differences between syncytia induced in γ-tubulin mutants, compared to the wild type (Figs. 3, 4), but they were sufficient to induce significant difference in the average numbers of females maturing on roots of γ-tubulin mutants and wild-type plants. Observed changes encompassed such important for effective feeding and development of juveniles features (Sobczak et al. 1997) as smaller sizes of syncytial elements, less cell wall openings, decreased syncytial cytoplasm density with increased number of vacuoles, more abundant formation of starch grains in plastids and the abundancy of concentric circular swirls of cisternal ER. In older developmental stages, syncytium commonly accumulates carbohydrates, such as starch grains in plastids that create a storage buffer to compensate changing nutritional demands of the developing juveniles (Hofmann et al. 2008). More numerous and larger starch grains in syncytia induced in γ-tubulin mutants might indicate that juveniles were not able to consume all the nutrients delivered to the syncytium. Another question is if the nutrients taken up to the syncytium can be efficiently delivered to the nematode. Syncytial cytoplasm is recognised by its high speed of streaming, especially in the region close to the nematode’s head (Wyss and Zunke 1986; Wyss 1992). Since the cytoskeleton is known to influence the cytoplasm fluidity and streaming, it might be supposed that modified MTs network in γ-tubulin mutants is unable to assure efficient speed of cytoplasm streaming responsible for nutrients delivery to the nematode. All these small differences indicating advanced physiological imbalance leading to premature syncytium senescence and disturbed development of juveniles became evident in 15 dpi syncytia formed in both γ-tubulin mutants. It has been reported that female nematodes consume 29 times more food than males and under prominent nutrient supply the majority of juveniles develops into adult females whereas under limited nutrients supply most of juveniles develop into males (Müller et al. 1982; Lilley et al. 2005). It seems that the lack of either of γ-tubulin isoforms might decrease also nutritional effectiveness of syncytia, which resulted in significantly lower number of developed females, but did not affected development of males.

During interphase in higher plants, γ-tubulins localize mainly to the cytoplasm, and to a lesser extent to the cortical MT arrays located close to the cell walls. Thus, recruitment of γ-tubulin complexes from the cytoplasm to the cortical MTs has been proposed. This agrees with previous data indicating that cytoplasmic MT arrays were fragmented in the syncytial cytoplasm, but present along the plasma membrane (de Almeida Engler et al. 2004), justifying the presence of MT nucleating proteins such as γ-tubulins. Based on the high degree of sequence identity, it was proposed that TUBG1 and TUBG2 play the same functions (Oakley and Akkari 1999; Pastuglia et al. 2006). However, our data showing distinct localization of γ-tubulin protein in syncytia of wild type and mutant lines as well as significantly reduced numbers of infecting juveniles and developed females in either of γ-tubulin mutants suggest that at least to a certain extent, they might fulfil distinct functions during cyst nematode feeding site development.

In 3 dpi syncytia, TUBG1 and TUBG2 were distributed uniformly over the syncytial cytoplasm and neighbouring vascular cylinder and cortex cells, while in 7 and 15 dpi syncytia, they were localized mostly in the paramural syncytial cytoplasm and next to the syncytial cell nuclei (Fig. 5). This distribution pattern generally conformed to the localization of mitochondria and plastids that were dispersed over the entire syncytial cytoplasm in young syncytia and usually located along cell walls in the paramural layer of syncytial cytoplasm in older syncytia (Fig. 4; Golinowski et al. 1996). Thus, it can be supposed that large organelles, such as mitochondria and plastids, are arranged along MTs arrays that create pathways for their trafficking. TUBG2 was localized additionally in the vicinity of syncytial nuclei which suggests that it might contribute to syncytial nuclei shaping or positioning as no mitotic activity was found in developing syncytia (de Almeida Engler et al. 1999). It can be speculated that the lack of one of γ-tubulin isoforms might discretely modify properties of the MTs leading to decreased efficiency of syncytium, e.g. via impaired sorting and localization of mitochondria and plastids accumulating starch grains. It might lead to shortage of nutrients delivered to the regions of syncytia adjacent the nematode’s head, and thus to disturbed development of female juveniles (Müller et al. 1982; Lilley et al. 2005). A distinct pattern of γ-tubulin localization was also observed in giant cells, where γ-tubulin was mainly localized around nuclei and to a lesser extent to cell cortex of giant cells and neighbouring cells (Banora et al. 2011).

Finally, we can implicate that observed changes in the numbers of invading juveniles and developing females in both γ-tubulin mutants were caused by disturbed development of plant cell walls. One of the most remarkable features of syncytium is prominent thickening of the outer syncytial cell walls whereas inner cell walls are locally dissolved and cell wall openings are formed (Jones and Northcote 1972; Golinowski et al. 1996; Sobczak et al. 1997). This process is the most obvious in older syncytia. In plant cells MT arrays create rafts anchoring cellulose synthase (Fisher and Cyr 1998; Wasteneys and Yang 2004; Lloyd and Chan 2008) and are pathways for delivery of hemicelluloses and pectin from Golgi apparatus to cell walls (Worden et al. 2012). It seems possible that the lack of one of γ-tubulins might disturb the pattern of cellulose fibrils deposition or reduce the amount or change the composition of other polysaccharides building cell walls. Modified pattern of plant cell walls could be responsible for observed changes in nematode infection rate as fewer juveniles could be able to recognise plant roots. It could be also responsible for lower number of cell wall openings and weaker hypertrophy of syncytial elements observed in syncytia induced in γ-tubulin mutants since modified syncytial cell wall could be more resistant to the action of cell wall degrading enzymes responsible for the formation of cell wall openings (Fudali et al. 2008; Karczmarek et al. 2008). Consecutively, lower number and smaller size of cell wall openings in syncytia induced in mutant plants might result in smaller region of confluent syncytial cytoplasm next to the nematode head that directly delivers nutrients to the juvenile (Wyss and Zunke 1986; Wyss 1992).

The present study does not provide clear and univocal explanations for the functions played by γ-tubulins in cyst nematode infection of *Arabidopsis* plants, but it is the first attempt to examine their role in syncytium and cyst nematode development. Herein, we show that although the expression of *TUBG1* and *TUBG2* genes changes from up-regulation during early stages of syncytium development till down-regulation in mature syncytia, the presence of both γ-tubulin isoforms has fundamental importance for nematode infection and female development. The lack of either γ-tubulin protein during syncytium induction, development and nematode maturation leads to deregulation of MT dynamics affecting the cytoskeleton in the feeding site. At early stages of syncytium development MT nucleation will most likely occur in the entire syncytial cytoplasm, whereas at later stages MTs are degraded in the cytoplasm, but remain in the cell cortex. This was also suggested by de Almeida Engler et al. (2004) and confirmed by our immunocytochemical analysis of TUBG1 and TUBG2 distribution. Moreover, taking into consideration the lack of TUBG1 and TUBG2 redundancy in infection tests, our novel results rise serious concerns if both γ-tubulin proteins do really play the same roles in plant cells.

### Author contribution statement

ER designed the experiments, performed most of experiments, analysed and interpreted the data, and wrote the manuscript. WC performed the quantitative qPCR analysis, carried out statistical analyses and helped to write the manuscript. ŁB performed immunolocalization studies. JM performed gel blots and immunodetection analysis. MS and JAE conceived the project, analysed data, helped to write the manuscript and critically revised it.

## Electronic supplementary material

Below is the link to the electronic supplementary material.

Supplementary material 1 (PDF 455 KB)

## Abbreviations

bp: Base pairs
dpi: Days post infection
J2: Second stage juvenile
MT: Microtubule
MTOC: Microtubule organizing centre
(RT)-qPCR: (Reverse transcription)-quantitative polymerase chain reaction
