# Supplementary material for: Expression of both Arabidopsis γ-tubulin genes is essential for development of a functional syncytium induced by Heterodera schachtii
Source: Plant Cell Rep. 2018 Jun 12;37(9):1279–92. doi: 10.1007/s00299-018-2312-7 (PMC6096582; doi:10.1007/s00299-018-2312-7)
Supplement: Supplementary file 1 — Supplementary material 1 (PDF 455 KB) [file 299_2018_2312_MOESM1_ESM.pdf]

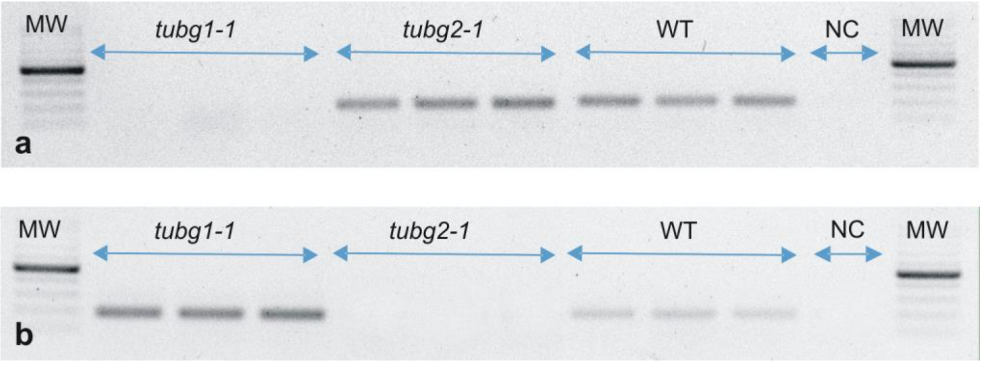

Fig. S1. Genotyping of wild-type and γ-tubulin mutant lines. (a) RT-PCR with primers amplifying *TUBG1* (Supporting Information Table S3); (b) RT-PCR with primers amplifying *TUBG2* (Supporting Information Table S3). Abbreviations: MW- molecular weight markers, WT- wild-type *A. thaliana* ecotype Col-0, NC- negative control.

```
  1 MPREIITLQVGQCGNQIGMEFWKQLCLEHGISKDGILEDFATQGGDRKDV     50
    ||||||||||||||||||||||||||||||||||||||||||||||||||
  1 MPREIITLQVGQCGNQIGMEFWKQLCLEHGISKDGILEDFATQGGDRKDV     50

 51 FFYQADDQHYIPRALLIDLEPRVINGIQNGDYRNLYNHENIFVADHGGGA    100
    ||||||||||||||||||||||||||||||:|||||||||||::||||||
 51 FFYQADDQHYIPRALLIDLEPRVINGIQNGEYRNLYNHENIFLSDHGGGA    100

101 GNNWASGYHQGKGVEEEIMDMIDREADGSDSLEGFVLCHSIAGGTGSGMG    150
    ||||||||||||||||||||||||||||||||||||||||||||||||||
101 GNNWASGYHQGKGVEEEIMDMIDREADGSDSLEGFVLCHSIAGGTGSGMG    150

151 SYLLETLNDRYSKKLVQTYSVFPNQMETSDVVVQPYNSLLTLKRLTLNAD    200
    ||||||||||||||||||||||||||||||||||||||||||||||||||
151 SYLLETLNDRYSKKLVQTYSVFPNQMETSDVVVQPYNSLLTLKRLTLNAD    200

201 CVVVLDNTALGRIAVERLHLTNPTFAQTNSLVSTVMSASTTTLRYPGYMN    250
    ||||||||||.|||||||||||||||||||||||||||||||||||||||
201 CVVVLDNTALNRIAVERLHLTNPTFAQTNSLVSTVMSASTTTLRYPGYMN    250

251 NDLVGLLASLIPTPRCHFLMTGYTPLTVERQANVIRKTTVLDVMRRLLQT    300
    ||||||||||||||||||||||||||||||||||||||||||||||||||
251 NDLVGLLASLIPTPRCHFLMTGYTPLTVERQANVIRKTTVLDVMRRLLQT    300

301 KNIMVSSYARNKEASQAKYISILNIIQGEVDPTQVHESLQRIRERKLVNF    350
    ||||||||||||||||||||||||||||||||||||||||||||||||||
301 KNIMVSSYARNKEASQAKYISILNIIQGEVDPTQVHESLQRIRERKLVNF    350

351 IEWGPASIQVALSKKSPYVQTAHRVSGLMLASHTSIRHLFSKCLSQYDKL    400
    |:||||||||||||||||||:||||||||||||||||||||:||||||||
351 IDWGPASIQVALSKKSPYVQTSHRVSGLMLASHTSIRHLFSRCLSQYDKL    400

401 RKKQAFLDNYRKFPMFADNDLSEFDESRDIIESLVDEYKACESPDYIKWG    450
    ||||||||||||||||||||||||||||||||||||||||||||||||||
401 RKKQAFLDNYRKFPMFADNDLSEFDESRDIIESLVDEYKACESPDYIKWG    450

451 MEDPEQLMTGEGNASGVVDPKLAF    474
    ||||.||||||||||||.||||||
451 MEDPGQLMTGEGNASGVADPKLAF    474

Aligned_sequences: 2
1: TUBG1
2: TUBG2
Matrix: EBLOSUM62
Gap_penalty: 10.0
Extend_penalty: 0.5

Length: 474
Identity:     465/474 (98.1%)
Similarity:   471/474 (99.4%)
Gaps:           0/474 ( 0.0%)
Score: 2430.0
```

Fig. S2. Comparison of amino acid sequences of *Arabidopsis* TUBG1 and TUBG2 proteins.

EEFATEGTDRKDVFFY peptide sequence (cat. no T6557; Sigma-Aldrich)
EDFATQGGDRKDVFFY *Arabidopsis* TUBG1
EDFATQGGDRKDVFFY *Arabidopsis* TUBG2

Fig. S3. Comparison of amino acid sequences of the oligopeptide used to generate specific γ-tubulin antibody with sequences of *Arabidopsis* TUBG1 and TUBG2 proteins.

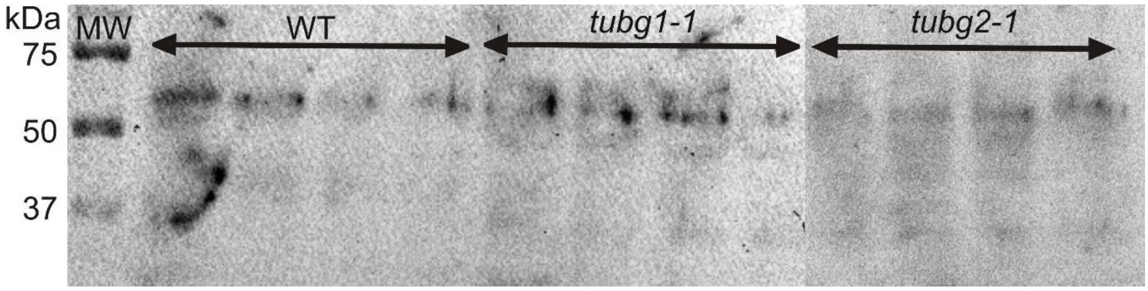

Fig. S4. Immunoblot analysis of anti-γ-TUBG antibody specificity carried on total protein extracts isolated from wild type Col-0 (WT) and mutant *tubg1-1* and *tubg2-1* plants. Abbreviations: MW, molecular weight markers (Precision Plus Protein™ All Blue Prestained Protein Standards; Bio-Rad, cat. no. #1610373).

Table S1. List of genes and sequences of primers used for real-time qPCR.

**Gene (ID)**	**Forward (F) and reverse (R) primer sequence (5'→3')**	**Product length [bp]**
AtTUBG1 (AT3G61650)	F: TCATGGACATGATTGATCGAG<br>R: TCCAACAAATAAGATCCCATACCT	112
AtTUBG2 (AT5G05620)	F: CAAATGGGGAATGGAGGAC<br>R: CTTAGGATCCGCAACTCCTG	73
AtUBP22(AT5G10790)	F: CACAAGGGGATGTTGGAATCAG<br>R:ACTCACATCCTCTCACCACTTC	121

Table S2. Program and conditions of real-time qPCR.

**Temperature**	**Time**
PCR	
50 °C	20 s
95 °C	10 min
40 cycles:	
95 °C	15 s
60 °C	60 s
Melting curve	
95 °C	15 s
60 °C	60 s
95 °C	30 s
60 °C	15 s

Table S3. List of genes and sequences of primers used for genotyping of γ-tubulin mutant lines by RT-PCR.

**Gene (ID)**	**Forward (F) and reverse (R) primer sequence (5'→3')**	**Product length [bp]**
AtTUBG1 (AT3G61650)	F: TCCTCACAGTCTCGAAACCC<br>R:ACATCTTTTCTATCACCTCCCTGA	171
AtTUBG2 (AT5G05620)	F: CCCAACTCCAAGGTGTCACT<br>R: AACATTTGCCTGCCAAGAAC	236

Table S4. Program and conditions of PCR.

**Temperature**	**Time**
PCR	
95°C	5 min
35 cycles:	
95 °C	30 s
55°C	30 s
72°C	60 s
72°C	10 min
